# Bioaccumulation of pesticides in fish resulting toxicities in humans through food chain and forensic aspects

**DOI:** 10.5620/eaht.2023017

**Published:** 2023-08-28

**Authors:** Suryapratap Ray, Sanjana Thanjan Shaju

**Affiliations:** Karunya Institute of Technology and Sciences, Tamil Nadu, India

**Keywords:** Bioaccumulation, Ecotoxicology, Fish, Water contamination

## Abstract

A crucial component for agricultural productivity is pesticide application. Increased usage of pesticides has significantly increased agricultural output, reduced grain losses in storage, and overall enhanced human wellbeing. Globally, every year approximately 3 billion kg of pesticides are used which budgets around 40 billion USD. Pesticide use can leave behind unwanted residues that can contaminate food, the environment, and living tissues. They are known to spread from agricultural regions that have been treated into the wider environment, where they affect non-target creatures. All tiers of biological organisms, directly impacted by this exposure. Pesticides at sub-lethal levels alter every aspect of a fish's physiology, including histology, haematology, defence mechanisms, and behaviour. The same topic of pesticide toxicology is the emphasis of this article, which also addresses some important induced chronic toxicological effects of pesticides in fish and the extent of their bioaccumulation in fish tissues. The data represents the largest bodies of water, such as rivers and lakes, that have been contaminated by pesticides, notably due to pesticide drift. It has been discussed how readily pesticides are absorbed into fish bodies and how this enters the food chain inducing harmful impacts on human health when consumed.

## Introduction

The study of aquatic toxicology examines how pollutants in the environment, particularly agrotoxins, industrial hazardous waste, toxic metabolites, etc., affect the health of fish and other aquatic organisms [1]. In other words, aquatic toxicology is the study of the effects of manufactured chemicals and other anthropogenic and natural materials and activities on aquatic organisms at various organizational levels, from subcellular to individual organisms to communities and ecosystems [1]. It is noted that pesticides used on crops are frequently drifted to the aquatic environment where they are frequently metabolized and bio-accumulated in aquatic food chains before being finally carried to humans. Pesticides absorbed by fish bodies cause them to move up the food chain and have harmful effects on human health upon consumption. Pesticide drift to water bodies causes chemical contamination, which has negative (chronic) impacts. Humans have been linked to cancer, obesity, endocrine disruption, and other disorders through exposure to pesticides and synthetic chemicals.

An agricultural region that has been artificially irrigated, such as by diverting streams, flooding, or spraying, is referred to as an irrigated agricultural area. Crop production in non-irrigated agricultural areas is reliant on rain-fed irrigation. A country's entire area, which may include terrain unsuited to agriculture such as woods, mountains, and interior waterways, only makes up a portion of its total area. Irrigated and non-irrigated land are additional categories for agricultural land. Agriculture is frequently restricted to irrigated land in dry and semi-arid regions, with very little farming feasible in unirrigated areas. Traditionally, in irrigated rice crops, a wide range of pesticides are frequently employed to manage pests, and fish are exposed to various pesticide concentrations and active components.

A study suggests that fish aquaculture in rice fields offers a way to simultaneously produce grain and animal protein on the same plot. Thus, in an age of environmental awareness, there are not many other food production systems that appear as efficient and environmentally sustainable. The term "rice-fish farming" suggests that rice and fish are farmed side by side in the same field while sharing the same water in separate plots. Most pesticides are toxic to a variety of nontarget organisms in addition to the target species since pest and non-pest species share structural and physiological characteristics. Non-target organisms include fish. The consequences of exposure to one type of pesticide at a time, such as organochlorine (OC), organophosphate (OP), or carbamate pesticides, have been the main subject of numerous ecotoxicology research involving fish [2]. Pesticides biomagnify along food chains and bioaccumulate in aquatic species. Through the food chain, this procedure may result in higher concentrations of pesticides and a possible impact on human health. The intake of fish might expose humans to accumulated pesticides [3]. Fish have been proven to have an impact on the nutrient makeup of floodwater, oxidise the soil surface, and have positive effects on rice growth, which makes them a vital part of the rice-nutrient cycle in rice fields. Fish provide nutrients to the rice fields through both their waste products and the decay of dead fish. Chemical pesticides are double-edged weapons that have the potential to harm both humans and the environment just as much as they may harm the target pests. As a result, eating fish that has been subjected to these pesticides can be considered a major cause of human exposure to environmental toxins [2]. When exposed to contamination stress, carp specimens experience considerable changes in their molecular, cellular, histological, and physiological characteristics. Fish are exposed to environmental contaminants through their gills, skin, and food. The bioaccumulation found in fish organs is used as a measure of the health of aquatic organisms [4]. The potential contamination rates brought on by pesticides used in rice cultures during the entire 100-day rice growth cycle because carp raised in integrated rice-fish farming are intended for human consumption. It was investigated whether these compounds bioaccumulate in the muscles of C. carpio, a species commonly used in integrated rice-fish farming, as well as the potential risks of these pesticides to freshwater fish (C. carpio) cultured in integrated rice-fish farming systems and their effects on the bodyweight, changes in the antioxidant defence system, and also the damage caused by oxidative stress. Based on the findings of the study, it is possible that humans who consume pesticide-contaminated fish run the risk of health problems due to the pesticide residues identified in C. carpio muscles. Changes in enzymatic and non-enzymatic antioxidants show that these pesticides cause oxidative stress. It was concluded that these pesticides were toxic to C. carpio even at levels that are permitted by law for use in rice cultivation. These pesticide combinations might vary between areas, primarily because of various environmental factors. Temperature, pH, and rainfall indexes are just a few examples of characteristics that vary with diverse climatic circumstances. These qualities are intimately related to the biochemical reactions produced in organisms exposed to various pollutants [2].

Water pollution is any alteration of a body of water's natural qualities brought on by anthropogenic contaminants to the point where it is unfit for human consumption or the sustenance of biotic organisms like fish, and other aquatic organisms. The term "water pollution" refers to the tainting of water bodies which includes lakes, rivers, oceans, and groundwater by human activity. Every type of water pollution has an impact on the flora and fauna that inhabit these bodies of water, and in almost all instances, this impact is detrimental to both the individual species and populations and the wider natural biological ecosystems [5]. It happens when contaminants are released into water bodies directly or indirectly without being adequately treated to remove dangerous components. Water pollution is a serious global health concern since it causes the emergence of several lethal diseases, which claim the lives of approximately 14,000 people every day. More concerning than the issue facing industrialised nations is the situation in developing nations [6].

Major determinants of survival, growth, reproduction, physiology and general behavior of fish are water quality parameters [7]. According to a study, most organic and inorganic pollutants cause a range of physiological and neurological disorders in humans and are carcinogenic and mutagenic. Numerous freshwater fish species accumulate more metals and pesticides from contaminated water than is permitted. Several hazardous substances, including methylated mercury and pesticides, have been observed to biomagnify in a variety of known Ganges fish. Consequently, over-accumulation of toxins in fish can be a significant source of exposure and consequent health risk to the fish-eating population.

The Ganga basin has seen an increase in cancer patients, with the biggest number of gallbladder cancer cases worldwide, according to the most recent data by the National Cancer Registry programme. The government has also focused on addressing the sewage issue, which is the biggest contributor to the pollution of the Ganga River. High levels of organic and inorganic contamination from industrial and agricultural effluents have been reported in several studies [8]. According to reports, many water contaminants behave like harmful chemical hazards. These are the pesticides which are not species-specific because they are made with the goal of killing pests and insects in general. They use application techniques that make sure the chemicals only target the intended pests, killing them while avoiding the non-targeted organisms. However, these target pests are merely animal species that exhibit a number of the same traits as other animals. Being vulnerable to specific chemicals is one of these traits. To put it in another way, a substance that is toxic to one species may also be toxic to other animal life. Many pesticides are harmful to humans even if it may require a higher dose to harm them than pests like insects. Humans are affected by the doses required to eradicate a pest in several ways, including disruptions to sex hormone functioning and reproductive efficiency [9-12]. Since the pesticides interfere with endocrine functions or operate as xenohormones (which resemble endogenous hormones), they are collectively categorised as endocrine disruptors [13].

Because the endocrine system is formed early in development, exposure to toxins can impair the development of endocrine tissue. The effects of OC (Organochlorine) exposure have been assessed in early developmental stages of many species. Chemical teratogenicity is studied in fish embryos. OC exposure during early development has been associated with mortality and deformities according to early studies, but recent research has attempted to understand how this exposure affects the neurotransmitter systems that control hormone release and brain development [14]. According to studies, several OCs can disrupt other endocrine systems, including those produced by the thyroid, and act as weak estrogens or antiandrogens in humans [15]. They have been connected to the onset of Parkinson's disease, and others, including dieldrin, have been identified as neuroendocrine disruptors that influence how the brain functions. These substances also impair immunological function [19,20], mitochondrial oxidative respiration [17,18], and regular metabolism [16,17]. OCs can therefore influence endocrine systems directly or indirectly by interfering with hormone synthesis, metabolism, and ATP generation [14]. In the aquatic ecosystem, fish are exposed to chemical pollutants through direct contact with their integuments, mouth, and gills by respiration. The most significant animal that provides food for humans is the fish. They are highly valuable from an economic, dietary, medical, industrial, aesthetic, and religious standpoint. Around the world, millions of people are provided employment because of fish cultivation directly or indirectly. They help to ensure food security and serve as an important addition to varied and nutritious diets. Protein, vitamins, and polyunsaturated fatty acids, which are believed to protect against cardiovascular disorders, are abundant in fish. Because they store a significant amount of nutrients in their tissues, transport nutrients further than other aquatic species, and excrete nutrients in dissolved forms that are easily accessible to primary producers, fish play a significant role in nutrient cycles in aquatic ecosystems [7].[Fig f1-eaht-38-3-e2023017]

## Pesticide

Organic pesticides or agrochemicals are by definition synthetic substances or a mixture of substances that are anthropogenically produced and applied to prevent, destroy or control undesirable species defined as fungal or animal pests. Consequently, these substances adversely affect the production, processing, storage, transport or marketing of several foods of plant or animal origin [21]. Pesticides and agrochemicals in general have become an important part of the world's agricultural systems over the last century, enabling significant increases in crop yields and food production [22]. Pesticides have been proven to be extremely noxious to aquatic life, including fish species and other aquatic organisms that are part of the tropical food web. Pesticides are used to manage damaging and invasive pests in agriculture, forestry and the landscape. They have the potential to enter the hydrological cycle at any point, and they can be transported long distances. The physical and chemical properties of a pesticide and the conditions of the environment to combine and determine if pesticides are going to persist in water and move to other locations. Pesticide-contaminated surface water is infamous for having an adverse effect on the aquatic and terrestrial ecosystems, with the toxicant moving from the lithosphere, hydrosphere, and atmosphere to harm the aquatic organisms' ability to survive and reproduce [23].

The occurrence of pesticide residues and their metabolites in various environmental compartments such as aquatic, air, soil and various foods is well documented, and several pesticides have been detected in various environmental matrices and biological tissues, usually at trace concentrations varying from ng L-1 to g L-1 [24]. For example, a body of published research data, followed by a significant number of related reviews [25-27], has demonstrated increasing scientific interest and global concern about the occurrence, fate and distribution of pesticides in the environment. The ability of a pesticide to hasten the negative effects of fish and aquatic species is significant. Its toxicity is usually influenced by factors like exposure period, dose rate, and environmental persistence.

The term "toxicity" describes how poisonous a pesticide is. While prolonged exposure to some chemicals may be harmful, brief contact may have a small impact on fish [23]. Pesticides are divided into four categories by WHO based on their level of toxicity: extremely dangerous, highly dangerous, moderately dangerous, and slightly dangerous [28]. Insecticides are the most toxic and acutely harmful of the three pesticides. They are classified into insecticides, which are used to control insects, herbicides, which are used to control weeds, and fungicides, which are used to treat mycotics. Finding ways to enhance and utilise safer pesticides to manage the broad spectrum of herb and insect pests that have a detrimental influence on the quantity and quality of global food supply has been one of the key supporters of the green revolution [29]. Organophosphate, carbamates, organochlorine, pyrethroids, and nicotinoids are a few of the different kinds of pesticides that are employed.

A study of two pesticides, malathion and diazinon, suggested their use as insecticides in paddy fields. They pollute the aquatic environment through direct application, drift, aerial spray and leaching from the atmosphere through precipitation, erosion and runoff from agricultural land, and through discharges into sewage, from factories and into sewers. Malathion has low toxicity to mammals and relatively high toxicity to fish. This happens due to the lack of hydrolytic enzymes in insects and fish. Oxygen analogs of malathion appear to be the active part that binds strongly to acetylcholinesterase. This malaoxon is rapidly hydrolyzed in mammals, rendering it inactive, but such hydrolysis does not occur in insects and is very slow in fish. Diazinon is a broad-spectrum insecticide [30]. Within a few weeks of the appliance, the water may get contaminated with chemical residues from intensive agricultural operations and drift. The use of insecticides slows growth rate and contributes to a variety of metabolic and reproductive disorders. It may result in histopathological changes in the gills, liver, hematopoietic tissues like the spleen, kidney, and renal tubules, in endocrine tissues as well as brain, neurological, and behavioural disorders when insecticides are exposed, particularly in fish species. It may also result in genetic defects. Some fish species are extremely vulnerable to water pollution from the environment [31].

## Classification of Pesticides

Pesticides are classified based on various criteria, including their toxicity (hazardous effects), the pest organisms they kill, their purpose, chemical makeup, their modes of entry and action, how or when they act, formulations, and sources of origin. Based on their chemical composition and the type of active substances they contain pesticides are categorized most frequently and most effectively. Such a classification offers details on the effectiveness, physical characteristics, and chemical composition of the relevant pesticides. To determine the method of application, the safety precautions to be taken when using them, and the application rates, it is very helpful to have knowledge of the chemical and physical features of pesticides. The classification of pesticides on a chemical basis is fairly intricate. Modern pesticides are typically organic compounds and comprise both synthetic and plant origin. Some inorganic substances are, nevertheless, also applied as pesticides. Insecticides are significant pesticides that fall into a number of subclasses [32].[Table t1-eaht-38-3-e2023017]

Although the purpose of pesticides is to destroy pests, this trait also renders them poisonous to other organisms, such as certain fish species, birds, mammals, and people. These pesticides don't have any particular targets and may become hazardous if they are continuously exposed to non-target species and cross the systemic threshold for toxicity. The majority of pesticides used in a region are known to find their way into healthy environmental elements like aquatic reserves (ponds, lakes, rivers, and oceans), where they eventually build up into other organisms. In the case of a pesticide, the active ingredient is the substance that has the ability to harm other animals while still being meant to kill the target insect. The toxin (active ingredient) is carried by the other chemicals in a pesticide, which are typically inert (not reactive) and utilised to facilitate application. Typically, just a very small portion of a pesticide's components are its active component. The capability of a pesticide to damage an exposed organism is referred to as its toxicity [35].

### Consequences of pesticide usage:

Pesticides have positive impacts on agricultural and public health, but they can also have negative consequences on the environment and on human health. Due to their high biological activity and toxicity, pesticides occupy a unique position among environmental pollutants. Most pesticides do not differentiate between pests and other accidental lifeforms that are similar to pests. If used improperly, they could be detrimental to people, animals, other living things, and the environment. Pesticides are thought to poison 500,000 to 1 million people annually and cause 5000–20,000 fatalities. Agricultural workers make up at least 50% of the intoxicated and 75% of the pesticide-related fatalities. Due to ingesting tainted food, the remainder is being poisoned [33].

### Indirect Effects of Pesticides on Fish:

Pesticides significantly limit the number of food organisms in aquatic ecosystems, which is essential for fish survival. By doing so, it alters the environment of water bodies and subtly disrupts the fish food supply. Additionally, by reducing habitat suitability and altering the fish's behavior, it may also render them more vulnerable to predators, which is a direct effect after an indirect effect. According to findings, these indirect impacts might often be far more critical than the direct ones [36].

### Direct Effects of Pesticides on Fish:

Pesticides have an immediate impact on fish. Pesticides cause a variety of toxicities in fish, including behavioral changes, haematological changes, histopathological disturbances, enzyme changes, genotoxicity, biochemical alterations, disruption of the endocrine system, variations in feeding biology, changes to the antioxidant defense system, and modifications to acetylcholinesterase activity. These chemicals can be toxic to many fish species in varying concentrations. It has been noted that the modifications in various bodily areas differ from one another and in reaction to certain pesticides. Nearly all systems and areas of the fish's body have shown these impacts [36].

### Potential risks on human health:

Pesticides can affect humans both directly and indirectly through a variety of pathways. However, food is the main source of direct human consumption of toxic substances. Vegetables and fruits grown on contaminated agricultural soils accumulate pesticides in their edible and inedible parts in concentrations high enough to cause clinical problems in animals and humans. Pesticides enter the human body through the skin, mouth, eyes and respiratory system, and hence scientifically confirmed acute diseases associated with pesticides include headache, abdominal pain, vomiting, rash, respiratory disorders, eye irritation, sneezing, convulsions and coma. Direct contact with pesticides can even lead to death. Oral exposure to pesticides is the key factor that determines their toxicity. Frequent consumption of foods based on agricultural crops grown on soils containing pesticides leads to short-term (acute) and long-term (chronic) diseases and disorders [37]. Pesticide runoff as a cause of water quality degradation has two significant negative effects on human health. The first is eating fish and shellfish contaminated with pesticides; This can be a particular problem for subsistence fisheries located downstream from large agricultural areas. The second is the direct consumption of water contaminated with pesticides [38]. Acute pesticide poisoning has now become a rare occurrence, but long-term subclinical effects remain a problem. Chronic toxicity caused by long-term exposure to low doses of pesticides can become evident much later. Chronic diseases include cancer, asthma, dermatitis, endocrine disorders, reproductive dysfunction, immunotoxicity, neurobehavioural disorders, and congenital defects. Chronic diseases can result from disruption of cellular homeostasis caused by the primary action of pesticides (disorders of enzymes, ion channels, and receptors; morphological changes in mitochondria) and accumulation of DNA damage [37]. Health Risk Assessment that consists of four parts, namely Hazard Identification, Dose–Response Assessment, Exposure Assessment, and Risk Characterization developed by the National Academy of Sciences in 1983 (National Academy of Sciences (NAS) 1983) can be used to determine the level of impairment to human health by chemical toxicity, exposure length, and intensity [39]. The nature of the poison, the mode of exposure (oral, cutaneous, and inhalation), the dose, and the organism all affect how toxic a chemical is. Insecticide toxicity is typically stated as a lethal dose (LD50) or lethal concentration (LC50). When a population is genetically homogeneous, the LD50 is the single exposure dose of the poison per unit weight of the organism needed to eliminate 50% of the test population. The measurement is in milligrams for every kilogram of body weight. When a population is genetically homogeneous, the LC50 is the chemical concentration in the external medium (often the air or water surrounding experimental animals) that results in 50% mortality of the test population. The measurement is in parts per million (ppm) [33].

### Adverse effects of pesticides on the Environment:

Studies indicate that a region is at risk of pollution when pesticide residues in the environment exceed no-effect levels, and at high risk when residues exceed this level by three orders of magnitude. It found that 64% of the world's agricultural land (approximately 24.5 million km2) is threatened by more than one active ingredient from pesticide exposures and 31% is at high risk. Of the high-risk areas, about 34% are in regions with high biodiversity, 5% in arid areas and 19% in low- and middle-income countries. Identification of watersheds in South Africa, China, India, Australia and Argentina as regions of particular concern due to their high risk of pesticide pollution, high biodiversity and suffering from water scarcity [40]. Pesticides can contaminate the ground, the water, the grass, and other vegetation. In addition to killing insects or weeds, they can also be toxic to other creatures such as birds, fish, beneficial insects, and non-target vegetation. Insecticides are typically the most toxic type of pesticide, but herbicides can also pose hazards to non-target creatures. Pesticide use in agriculture could contaminate surface waters through runoff, runoff, runoff and leaching [41]. Limiting the insecticides' effect region is practically impossible. Even when applied in a relatively small space, it gradually travels through the air, absorbs into the ground, or dissolves in water to reach a much larger region. Once discharged into the environment, pesticides may have a variety of outcomes. The contaminated surface water adversely affects the living organisms as the directly applied pesticides to the soil have the potential to wash off, percolate through the soil to lower soil layers, and reach neighboring surface water bodies through surface runoff [42]. In developing countries, surface waters serve as the main source of drinking water. Pesticides' effects on the environment might range from slight changes in the ecosystem's proper functioning to the extinction of some species. When using pesticides, there are occasionally long-lasting side effects in addition to acute fatal consequences. For instance, the majority of organochlorine pesticides are long-lasting in the environment and contaminate groundwater, surface water, food products, air, and soil [33].

### Impact of pesticide usage on Aquatic Ecosystem:

Pesticide pollution can occur when agricultural, industrial, or commercial wastes are disposed of in an aquatic environment. Some of these chemical compounds can persist in soil and sediment for years before entering groundwater systems or volatilizing into the atmosphere. Since runoff has an affinity for sediments and drainage systems, pesticides are often persistent in the environment and their main sinks are rivers [7]. Pesticides can infiltrate water bodies through diffuse (non-point) or direct (point) sources. The non-point source is the movement of pesticides from large areas across the watershed and eventually reaches the water bodies over time. Non-point sources of pesticides come from the agricultural field triggered by runoff and erosion events, resulting in the gradual leaching of pesticides into ground and surface water. These include the following: tile drain outflow, baseflow seepage, surface and subsurface runoff, and soil erosion from treated fields; as well as spray drift during application and deposition following volatilization. On the other hand, point source originating from a fixed location, including chemical discharges during improper storage, loading, disposal, and misapplication of pesticides in water bodies. Direct discharge of pesticides into groundwater is a common mode of point source pollution where the pesticides enter water wells as a result of pesticide spills and improper disposal of pesticides. Urban use of insecticides is considered as a point source pesticide in surface waters. These primarily include sewage plants, sewer overflows, farmyard runoff, and unintentional spills. Additionally, there are point sources of pesticides used in non-agricultural settings, such as when they are applied on sealed urban surfaces like parking lots, roads, or railroads [43]. These harmful compounds get into the water sources through a variety of different channels, including spills, industrial effluent, surface runoff, or soils that have been exposed to pesticides. According to the exposure length, which can be short or long-term, and exposure type, which can be fatal or sub-lethal, the toxic consequences brought on by exposure to these toxic substances can be divided into different categories where short-term exposure is defined as lasting less than 96 hours, whereas long-term exposure is defined as lasting longer than 96 hours. Pesticides from agricultural fields typically run off to reservoirs or drainage systems during heavy rain or during irrigation. Pesticides primarily reach aquatic organisms in three ways: Pesticides can harm aquatic organisms in three ways: (i) through the skin; (ii) through breathing; and (iii) orally. Aquatic organisms are typically exposed to pesticides either by feeding on pesticide-contaminated prey or by drinking pesticide-contaminated water. As aquatic organisms are in direct contact with water, pesticides can harm them through dermal pores [35].

## Concept of Bioconcentration, Bioaccumulation and Biomagnification

When assessing the actual environmental consequences of pesticides, aquatic organisms' interactions with one another and their propensity to bioconcentrate residues in water and via dietary intake are crucial to consider. The process by which pesticides enter organisms directly from water through the gills or through epithelial tissues is most commonly referred to as "bioconcentration." Bioaccumulation, on the other hand, takes into account the impact of nutritional absorption through food consumption or ingestion of bottom sediments. Biomagnification is the process by which pesticide levels collected by organisms are concentrated via two or more trophic levels [44]. The term describes the increasing content of a chemical in food energy that is converted within the food chain. Up the food chain, fewer living organisms are eaten by larger organisms, gradually increasing levels of pesticides and other chemicals in tissues and other organs. A very high level can be observed in higher predators, including humans [45-48].

Studies showed that different fish tissues had accumulated pesticides and the extent of accumulation varied widely from one pesticide to another. In general, bioconcentration is influenced by the structure of the compound, its water solubility, the physiological activity of the animal, and other vital factors such as temperature, water organic matter content, and population density. The term defines the transfer of a chemical into an organism from the surrounding medium. It is known that adipose tissues (lipids) are the main receptor for many insecticides, so these pesticides accumulate in fats, and an example of these pesticides is DDT. DDT is lipophilic because it is fat-soluble in human adipose tissue when edible fish and edible fish tissue contaminated with DDT are consumed [49,50]. One of the important features to emerge from the studies is that bioaccumulation was relatively low for all pesticide concentrations tested and the degree of accumulation differed from fish to fish. This clearly shows that species-specific factors play an important role and chemical properties of pesticides determine the outcome of bioaccumulation. Bioaccumulation was maximal when the exposure time was maximal.

A study proved that liver tissue was the one showing greater accumulation, followed by kidney, muscle tissue, gills and finally the intestine. Among the various organs, the liver and muscle are frequently examined for residue accumulation. Most literature shows that the residue concentration is higher in muscle than in liver tissue.

## Oxidative stress caused by pesticides

Through an increase in oxidative stress, pesticides have an impact on human metabolism. Oxidative stress is defined as a consequence of an increased level of reactive species and a reduction in the physiological antioxidant defenses against them. Pesticide activity in humans has been linked to endocrine disruption and an increase in oxidative stress levels. Exposure to pesticides plays an important role in increasing oxidative stress levels and may result in altered disease susceptibility. They have been identified in the human placenta and caused lower birth weight, intrauterine growth restriction and increased oxidative stress. In particular, lipid peroxidation (as one of the main oxidative stress parameters) and DNA damage have been observed in humans, mainly agricultural farmers and pregnant women. However, there is very little research on mammals using pesticides that directly link pollutant toxicity and oxidative stress. Therefore, studies with human cell lines began, which are now being carried out on a larger scale. Using human cell lines, changes in hormonal balance and the effects of pesticides on oxidative stress levels in human cells have been demonstrated [51].

Fish can thrive in a wide range of aquatic settings around the world, including those with varying temperatures, salinities, and levels of dissolved oxygen. In aquatic organisms, oxidative stress is induced by multiple actions of different factors in suspended matter and sediments. Climate change is leading to a hypoxic environment in water, meaning oxygen concentrations below 2 mg/L. Temperature conditions can also alter absorption and sensitivity of response to exposure to external factors. Higher water temperatures decrease the amount of dissolved oxygen, which increases the oxygen demand of the fish. Oxygen limitations lead to biochemical adaptations to avoid or resist stress in aquatic organisms. Excessive heat stress conditions induce synergies in the action of pollutants that alter the oxidation mechanism in fish. Oxidative stress is caused by disruptions in free radical production and antioxidant activity. Oxidative stress is considered to be the main mechanism of non-targeted action of pesticides and poses risks of neurotoxicity, cardiovascular toxicity and reproductive toxicity depending on the organs in which it occurs. Sperm quality is an important factor in assessing reproductive function in male fish. Fish sperm cells are released into the aquatic environment and are directly exposed to several pollutants prior to fertilization. The occurrence of oxidative stress in sperm affects sperm motility and disrupts their membranes and DNA integrity [52].

A study showed that the recorded pesticide concentrations and evidence of bioaccumulation in muscle tissue could explain the biochemical changes that occurred in the following parameters: AChE, CAT and GST activity, TBARS and PC levels, and NPSH and AsA levels. Changes in these parameters reflect the oxidative damage, responses, and mechanisms by which organisms protect themselves against the toxicity of pollutants.

The phenomenon of lipid peroxidation was indicated by increased levels of TBARS in liver, muscle and gills, providing an indication of lipid damage as a result of the combined use of pesticides. Such results may stem from variations in the antioxidant mechanisms found in fish species. These mechanisms are triggered by exposure to different classes of pesticides as they occur under real field conditions. These elevated levels of TBARS may result from impairments in antioxidant enzymes caused by reactive oxygen species (ROS) formation. The PC content was increased in the tissues tested and the protein damage detected was possibly caused by increased concentrations of pesticides in the fish organisms, but also by molecules resulting from oxidation processes such as MDA, or by direct ROS attack on their structure. Taken together, PC levels coupled with TBARS levels could explain the oxidative damage caused by combined exposure to pesticides. Most of the available scientific information is based on exposure to individual pesticides, so we can conclude that when combined with other pesticides, the damage caused could increase. The increased and decreased CAT activity indicates a disruption in the normal oxidation process, suggesting a failure of the antioxidant defence system. GST activity was higher in liver, gills and brain, but no changes in muscle tissue were observed when the experimental group was compared to the control group. Induction of antioxidant enzymes, such as CAT and GST, may be an important adaptation to pollutant-induced stress caused by exposure to pesticides. Lipid peroxidation is one of the factors associated with an increase in GST activity. Some studies have shown a relationship between increased GST activity and a decrease in TBARS levels [2].[Fig f2-eaht-38-3-e2023017][Table t2-eaht-38-3-e2023017]

### LC50 Value of Some Pesticides for Different Species:

It has been suggested that the toxicity of a pesticide can be modified by several factors, including the physicochemical properties of the medium and the biological behavior and status of the test animal. The status of the test fish includes size, weight, age, sex and life cycle stage. The physicochemical factors such as temperature, pH, alkalinity and hardness also influence toxicity. Effects on fish can be transmitted to other trophic levels and cause a wider range of damage. The acute toxicity test provides a quick and inexpensive method of measuring relative toxicity in different water species. Toxicity is the property of an individual organ's response to a chemical at a specific concentration or dosage for a specific period of time. The use of LC50 values is well established among toxicologists and is generally the most widely used test for assessing the potential pre-effect of aquatic life. LC50 values differ from species to species for the same pesticide and different pesticides due to their mode of action. The toxicity curve is generally plotted to understand how pesticides work. The shape of the curve indicates the type of pesticide effect, which is either cumulative or regular or irregular. LC50 values are a useful measure of the acute toxicity of tested pesticides used in specific environmental conditions, but do not truly represent a concentration that may be safe or benign for fish habitats exposed to contamination [55]. Acute and sublethal toxicity tests are commonly used to assess the toxicity of chemicals to non-target animals. The 96h LC50 is one of the most important factors in evaluating the toxic effects of pollutants [56].

In a study, after being treated to the pesticide Elsan for 48 hours, the test organism Channa punctatus produced an LC50 value of 0.43 ppm and for the pesticide Diazinon after an exposure of 96 hours, a value of 3.09 ppm. The organism Cyprinus carpio similarly demonstrated individual values of 35 µg/L and 4.21 mg/L following 24-hour exposure to Permethrin and Biosal, respectively, as well as a value of 0.160 µg/L after 96 hours of treatment to the pesticide Karate. The test organism, Labeo rohita, was exposed to Cypermethrin, Malathion, Endosulfan, Dimethoate, and λ Cyhalothrin over the course of 96 hours, yielding LC50 values of 4.0 µ/L, 15 mg/L, 2.15 µg/L, 24.55 µg/L, and 0.7 µg/L, respectively. Salmo gairdneri has levels of 2.4 mg/L and 8.7 µg/L following 96 hours of exposure to Alaclor and DDT, correspondingly. After 96 hours of exposure, Catla catla offers the pesticides Methyl parathion and Endosulfan values of 4.8 ppm and 0.98 µg/L, respectively. Ictalarus punctatus, Channa striatus, and Cirrhinus mrigala were subjected to endosulfan for 96 hours, and their respective results were 1.5 µg/L, 0.0035 ppm, and 1.06 µg/L. Diazinon produced results of 6.55 ppm and 2.72 ppm, respectively, after being exposed for 4 days to the test organisms Anabas testudineus and Barbodes gonionotus. Similar results were obtained when Channel catfish were exposed to Akton for 96 hours, yielding an LC50 value of 400 µg/L, and when Carassius auratus was exposed to BHC, producing a value of 348 µg/L. After being exposed to the organisms Salvelinus namaycush, Perca flavescens and Pimephales promelas, carbaryl, carbofuran and Acephate generated findings of 690 µg/L, 147 µg/L, and >1000 mg/L respectively. Additionally, Danio rerio, Colisa fasciatus, Nemacheilus botia, Puntius stigma, and Clarias gariepinus were exposed to pesticides such as λ Cyhalothrin, Cypermethrin, Metasystox, Rogor, and Termifos for 4 days. These results showed that the LC50 values were 0.119 µ/L, 0.02 mg/L, 7.018 ppm, 7.1 ppm and 0.86 mg/L [36].[Table t3-eaht-38-3-e2023017]

Samples of persistent pesticides concentration levels and occurrence patterns were taken from the river Tapi in Gujarat, India. This river was examined for the presence of chlorpyrifos, methyl parathion, DDT and endosulfan. Surface water samples revealed amounts of 0.03756ppm, 0.00086ppm, and 0.00043ppm for endosulfan, chlorpyrifos, and methyl parathion, respectively. Sediment had 0.03838ppm of endosulfan, and 0.00077ppm of methyl parathion. Fish samples from various locations were found to contain significant amounts of endosulfan, chlorpyrifos, and methyl parathion, with concentrations of 0.10128, 0.000392, and 0.00349 ppm, respectively [57, 58].

In the Terai region of West Bengal's Deomoni river, chlorpyriphos, ethion, and dicofol were also found in water, sediment, and fish at varying concentrations, as determined by statistical analysis. The levels of pesticide residues were found to be highly significant (p<=0.001) in all three types of samples tested, except for dicofol in water and sediment, which was significant at p<=0.01 [19].[Table t4-eaht-38-3-e2023017]

Samples of persistent pesticide concentration levels and occurrence patterns were taken from the river Tapi in Gujarat, India. This river was examined for the presence of chlorpyrifos, methyl parathion, and endosulfan. Surface water samples revealed amounts of 0.0376ppm, 0.0009ppm, and 0.0004ppm for endosulfan, chlorpyrifos, and methyl parathion, respectively. Sediment had 0.03838ppm of endosulfan, 0.00065ppm of Chlorpyrifos, and 0.00077ppm of methyl parathion, respectively. Endosulfan, chlorpyrifos, and methyl parathion were found in fish samples at concentrations of 0.10128, 0.000392, and 0.00349 ppm, respectively [57]. Chlorpyriphos, ethion, and dicofol analyses in the Terai region of West Bengal's river Deomoni revealed mean pesticide residues on the water surface of 0.0091 ± 0.0020 ppm, 0.0892 ± 0.0375 ppm, and 0.0180 ± 0.0071 ppm respectively; in sediments, 0.0513 ± 0.0085 ppm, 0.1271 ± 0.0122 ppm and 0.0717 ± 0.0075 ppm and in fish body, 5.0371 ± 1.7236 ppm, 2.9599 ± 0.4027 ppm, and 3.7700 ± 0.6391 ppm respectively. With the exception of dicofol in water and sediment, which is significant at p<=0.01, the analysis of pesticide residues by one way ANOVA for chlorpyriphos, dicofol, and ethion in the water and sediments, the sediment and muscles, and the water and muscles was found to be highly significant (p<=0.001) [58].

Comparing the data in water and sediment revealed higher concentrations of the pesticides in fish muscle than in sediment and water, while water had the lowest concentration. The lower levels of pesticide residues in the water bodies than in the sediment could be attributed to the fact that the input of pesticides into the water depends on seasonally different concentrations of suspended matter into which the residues were taken up and transported. dependent on the precipitation events that drive soil erosion activities and suspended solids levels during runoff. Higher levels of pesticides were found in the sediments than in the water because sediments are important sinks for various pollutants such as pesticides, which under favourable conditions also play a significant role in pollutant remobilization in aquatic systems and water-sediment interactions. Sediments act as a secondary source of contamination after water in the ecosystem. Sediments are the main reservoirs of environmental pesticides and provide a source from which residues can be released into the atmosphere, groundwater and living organisms. The higher levels of pesticides found in the fish could be due to the fact that fish have a greater tendency to accumulate the pesticides in their bodies due to bioaccumulation [58].

### Forensic Aspects:

One case reported the toxic effect of endosulfan on the development of some males. In Kasargod, southern India, where villagers were repeatedly exposed to aerially sprayed endosulfan (a banned pesticide), the boys suffered from delayed sexual development (markedly reduced development of pubic hair, testicles and penis), reduced testosterone synthesis and high levels of luteinizing hormone [59]. The maximum level of total DDT (21.81 ppm) was found in human fat samples collected in Ahmedabad. While BHC (benzene hexachloride) content was 147.93 µ g/l, which ranged from 34.66 to 231.47 µ g/l, pp-DDE and total DDT in serum samples ranged from 8.64 to 137.26 and 10.34164.2 µ g/l. In one of the studies, a total of 116 cases of suicide due to OPP poisoning were reported at the New Civil Hospital and Government Medical College in Surat, Gujarat. Pesticides called Monocrotophos (MCP), Methylparathion and Quinalphos were used for suicide attempts [60].

Until it was outlawed in 1990, the organochlorine insecticide Endrin caused several fatalities in the 1960s and 1980s. Endosulfan was a big problem thereafter, until it was also banned in Kerala in 2005 and across India in 2011; The impact of this ban is not apparent from the data as the number of reported cases has remained fairly constant over the past 10 years. The illegal importation of some endosulfan, despite the ban, could be partly to blame. Deaths from organochlorine insecticides are still being reported in some states of India (Rajasthan, Andhra Pradesh, Karnataka, Tamil Nadu). The impact of India's Supreme Court driven ban on endosulfan should be carefully studied, although initial analysis using national police data suggests it may have been linked to a nationwide decline in pesticide-related suicides. relatively few deaths were found from herbicides, including paraquat; they were responsible for only 1.8% of all reported pesticide-related deaths. This contradicts claims that herbicides were commonly used in suicide attempts in India. However, there has been an increasing trend of paraquat poisoning in India. A hospital study conducted from 1999 to 2006 reported five patients (three deaths) in Punjab, while a second from Tamil Nadu reported ten patients with 100% mortality. According to a recent newspaper report, paraquat poisoning is a problem in Orissa with more than 100 deaths reported in 2018 and 2019. If paraquat is used extensively in India for agriculture and self-harm, there is a risk of a massive spike in deaths, as was the case in China before its ban in 2016 [61]. Of forensic importance, the river water contaminated with pesticides can lead to chronic poisoning or deformities in the people living around the river since river water is the water source used by them for their daily lives. This can result in severe consequences and in many cases lead to death due to regular consumption of the contaminated water. In addition, due to its regular consumption, it causes the bioaccumulation of banned pesticides in the human body [57].

## Conclusions

Studies so far suggested different aspects of pesticide drift and its impact towards the surrounding and ecosystem. It can be seen that improper use of pesticides can adversely affect all levels of biological organization and every component of the environment. Previous studies indicate that pesticides may cause toxic effects and structural changes in non-target organisms such as fish. A concerted effort is needed to reduce the use of chemical pesticides. In addition, regular biomonitoring to know the hazards they pose, there is a need for research that would provide early warning signals for the contamination of certain pesticides in aquatic systems. The current review gathered valuable evidence suggesting multiple areas that are adversely affected by such human activity. In one of the recent studies, The river Tapi in Gujarat was examined and the surface water samples revealed the concentration of 0.0376 ppm, 0.0009 ppm, and 0.0004 ppm for endosulfan, chlorpyrifos, and methyl parathion, respectively; the sediment had 0.03838ppm of endosulfan, 0.00065 ppm of Chlorpyrifos, and 0.00077ppm of methyl parathion, respectively; finally, fish samples had concentrations of endosulfan, chlorpyrifos, and methyl parathion found at 0.10128, 0.000392, and 0.00349 ppm, respectively. Chlorpyriphos, ethion, and dicofol analyses in the Terai region of West Bengal's river Deomoni revealed mean pesticide residues on the water surface of 0.0091 ± 0.0020 ppm, 0.0892 ± 0.0375 ppm, and 0.0180 ± 0.0071 ppm respectively; in sediments, 0.0513 ± 0.0085 ppm, 0.1271 ± 0.0122 ppm and 0.0717 ± 0.0075 ppm and in fish body, 5.0371 ± 1.7236 ppm, 2.9599 ± 0.4027 ppm, and 3.7700 ± 0.6391 ppm respectively. Waterbody contamination all over the world induces ill effects to the fish and are constantly monitored for safety aspects providing acceptable contamination levels and so on. Studies by different researchers as per the available literature suggest the most impacted organ in fish body and the most frequently observed symptoms of toxicity. Among the various organs, the liver and muscle are frequently examined for residue accumulation. Most literature shows that the residue concentration is higher in muscle than in liver tissue. The enzymatic and non-enzymatic parameter-based studies considering oxidative stress suggested the bioaccumulation of agrotoxins in fish bodies and the low acetylcholinesterase activity in CNS level. In different organ levels, the Lipid peroxidation concentration was found to be in peak as well as the Protein oxidation higher. One of the major balanced nutrient rich food sources i.e., Fish is consumed in India’s almost all parts and has proven to be a vector of toxic elements as the literature suggests. Studies at the national and regional levels in order to keep the monitoring process on track are needed in this critical area. The frequent updates regarding contamination levels of water bodies and bioaccumulation will keep the regulating authorities aware and produce the guideline as per the information generated through research.

## Figures and Tables

**Figure 1. f1-eaht-38-3-e2023017:**
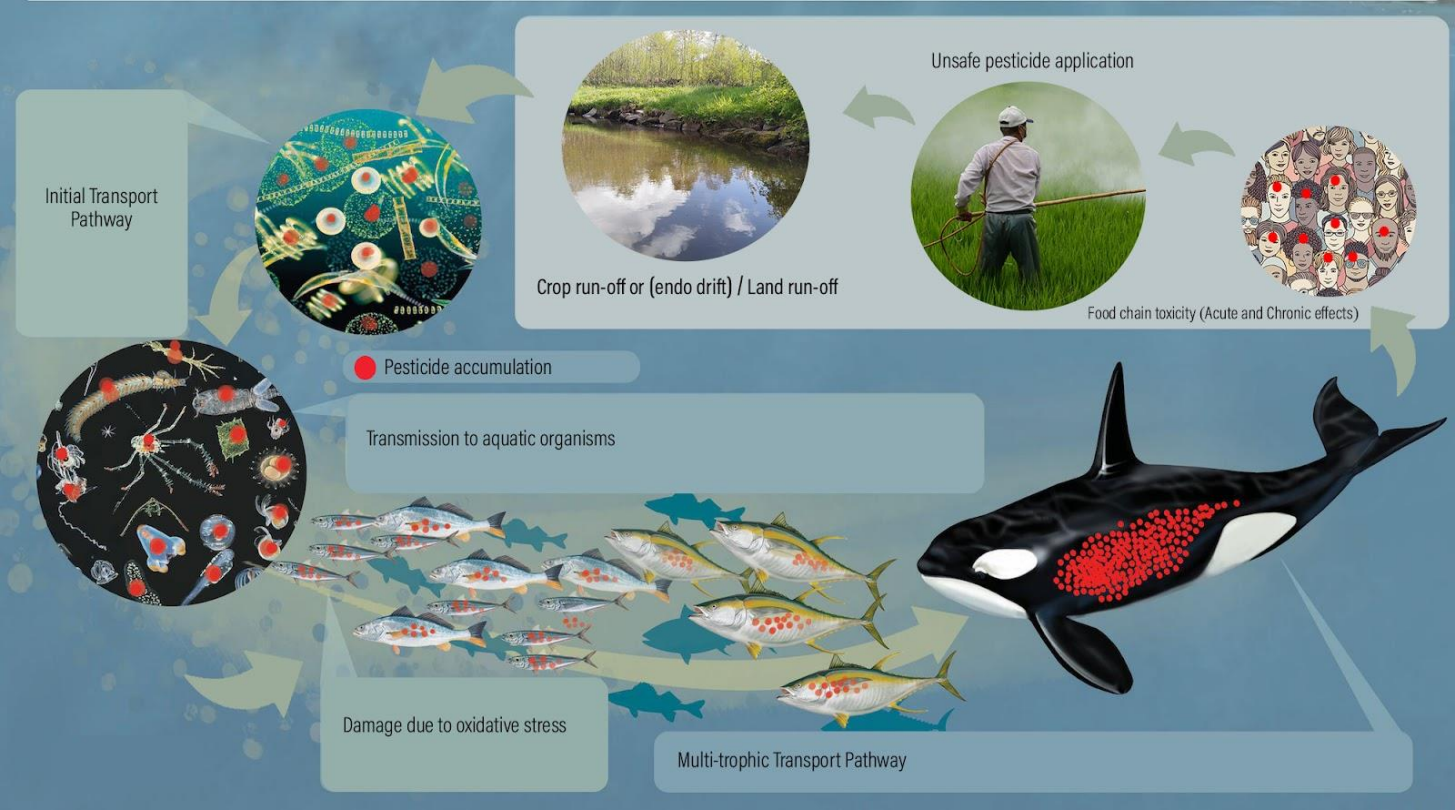
Collateral effects of toxic pollutants on aquatic organisms and its effects on humans

**Figure 2. f2-eaht-38-3-e2023017:**
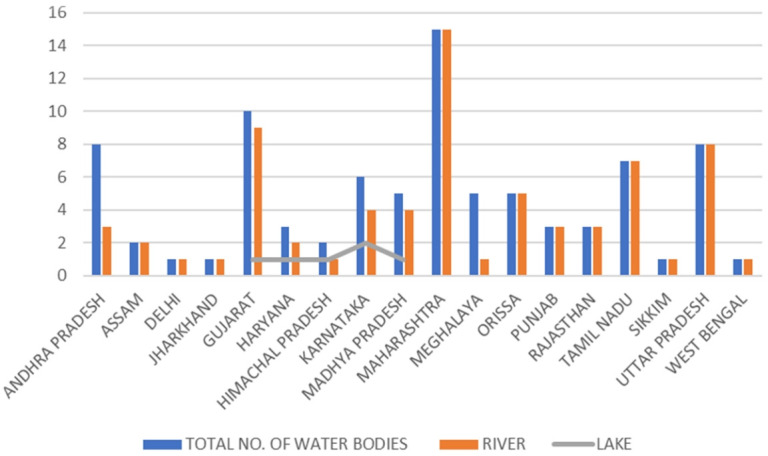
State-specific contaminated stretches in Indian rivers and lakes [53]

**Table 1. t1-eaht-38-3-e2023017:** Classification of Pesticides based on (i) the mode of entry, (ii) the action, (iii) the chemical composition, (iv) the toxicity, and (v) the source of origin

Classification Based on	Class	Sub-class	Examples	Use/Function	Reference
Mode of entry	Systemic pesticide		2,4-D, glyphosate	They can kill weeds in addition to being absorbed by plants or animals and moving to untreated tissues.	
	Non-systemic (Contact)		Paraquat, diquat	They act on target pests when they come in contact.	
	Stomach		Malathion	They enter into the pest’s body through their mouth and digestive system.	[32-34]
	Fumigant		Phosphine	They act or may kill the target pests by producing vapour and entering the pest body through a tracheal system.	
	Repellent		Methiocarb	They do not kill the pest’s but make pesticides target tasteless enough to keep pests away from the treated area	
Action	Physical Poison		Activated clay	Starts killing pests by applying a physical effect	
	Protoplasm		Arsenicals	Subjected for the precipitation of protein	
	Respiratory		Hydrogen cyanide	Inactivate the respiratory enzymes	
	Nerve		Malathion	Obstruct impulse conduction	
	Chitin Inhibition		Diflubenzuron	Obstruct the chitin synthesis in pests	
Chemical Composition	Insecticide:	Organophosphates	Monocrotophos	Kill or repel insects and related species	
		Carbamates	Carbaryl		
		Pyrethroids	Permethrin		
		Organochlorines	Endosulfan		
		Neonicotinoids	Imidacloprid		
		a) Fungicides:	Dodine	Used to prevent and cure to completely eliminate the fungi	
		Aliphatic nitrogen fungicides	Carpropamid		
		Amide fungicides			
		Aromatic fungicides	Chlorothalonil		
		Dicarboximide fungicides	Famoxadone		
		Dinitrophenol fungicides	Dinocap		
		b) Herbicide:	Flufenacet	Used to kill weeds or obstruct the growth of undesirable herbs or weeds	
		Anilide herbicides			
		Phenoxyacetic herbicides	2, 4-D		
		Quaternary ammonium herbicides			
		Chlorotriazine herbicides	Paraquat		
		Sulfonylurea herbicides			
			Atrazine		
			Chlorimuron		
		c) Rodenticide:		Used to kill mice and other rodents	
		Inorganic rodenticides	Zinc phosphide, Aluminium Phosphide		
		Coumarin rodenticides			
			(organic) Bromadiolone, Coumatetralyl		
Toxicity	Acute		Carbamates (Carbaryl, Thiram)	Indicate how poisonous a pesticide for human, animal or plant is after a single short-term exposure	
			Organochlorines (Aldrin, lindane)		
			Organophosphates (Malathion, Parathion, Chlorpyrifos)		
	Chronic		Organochlorines (DDT, Endosulfan)	Indicate how poisonous a pesticide for human, animal or plant is after a regular dose long-term exposure	
Source of Origin	Biopesticide:		Azadirachtin (Neem bio-pesticide)	Acts on the target pests and those organisms which are strongly linked to the pests	
		a) Microbial	Bacterial toxins	Made up of microorganisms and the active component of these microbial pesticides are microorganisms	
		b) Plant incorporated protectants	GMO with foreign genes	They are produced by plants naturally and genetic material introduced together	
		c)Biochemical pesticides	Insect sex pheromones	They combine natural material that have non-toxic mechanism to controls pests	
	Chemical	a) Organochlorine		Toxic and not constantly biodegradable and also affects a large group of non-target organisms	
		b) Organophosphate			
		c) Carbamates			
		d) Pyrethroids			

**Table 2. t2-eaht-38-3-e2023017:** Data suggesting the contamination level of major rivers in India due to pesticides and the standard guidelines

Pesticide	Rivers of India				Standard Guidelines					
	Brahmaputra River	Chilika Lake	Tapi River	Hooghly River	USEPA, AV	WHO, GV	USA, MCL	AUS, HV	NZ, MAV	Reference
Aldrin	0.005	-	-	0.009	0.36	0.03	-	0.03	-	
Deldrin	0.019	-	-	0.007	0.36	0.03	-	0.03	-	[54]
Chlorpyrifos	-	2.73	0.27	-	0.083	-	2.0	-	70.0	
DDTs	0.225	23.58	3.15	0.026	1.10	2.0	-	20.0	2.0	
Endosulfan	0.053	-	14.18	0.010	0.22	-	0.20	30.0	-	
Heptachlor	0.010	1.00	-	0.026	0.52	0.03	0.40	0.30	0.04	
HCHs	0.022	6.60	-	0.114	2.00	-	0.20	20.0	2.0	

**Table 3. t3-eaht-38-3-e2023017:** Characteristics of some insecticides with respect to their relative toxicity to fish

INSECTICIDE	RELATIVE RUN-OFF POTENTIAL	RELATIVE LEACHING POTENTIAL	HALF LIFE IN DAYS	RELATIVE TOXICITY TO FISH	REFERENCE
Hydramethylnon	Large	Small	10	High	[19]
Diazinon	Medium	Large	30	High	
Chlorpyrifos	Large	Small	30	Very High	
Malathion	Small	Small	1	Very High	
Acephate	Small	Small	3	Very Low	
Carbaryl	Medium	Small	10	Medium	
Dimethoate	Small	Medium	7	Medium	
Trichlorfon	Small	Large	27	High	
Dicofol	Large	Small	60	High	
Propargite	Large	Small	56	High	

**Table 4. t4-eaht-38-3-e2023017:** Comparison of the values of pesticides in the water, sediment and fish muscle from river Tapi, Gujarat and Deomoni, West Bengal

	Quantity of Pesticide in Water and Fish (ppm)					Reference
	Endosulfan	Chlorpyrifos	Methyl parathion	Ethion	Dicofol	
Surface water	0.0376	0.0009	0.0004	0.0892 ± 0.0375	0.0180 ± 0.0071	[57, 58]
sediment	0.03838	0.00065	0.00077	0.1271 ± 0.0122	0.0717 ± 0.0075	
Fish muscle	0.10128	0.000392	0.00349	2.9599 ± 0.4027	3.7700 ± 0.6391	
